# Sexual transmission of Zika virus enhances *in utero* transmission in a mouse model

**DOI:** 10.1038/s41598-018-22840-6

**Published:** 2018-03-14

**Authors:** Nisha K. Duggal, Erin M. McDonald, Jana M. Ritter, Aaron C. Brault

**Affiliations:** 1 Division of Vector-borne Diseases, Centers for Disease Control and Prevention, Fort Collins, Colorado, USA; 2 Division of High-Consequence Pathogens and Pathology, Centers for Disease Control and Prevention, Atlanta, Georgia

## Abstract

Zika virus (ZIKV) is an emerging mosquito-borne virus that can cause ZIKV congenital syndrome when a pregnant woman is infected. Sexual transmission has also been described for ZIKV, though the relationship between sexual transmission and vertical transmission has not been investigated. Here, viral dissemination to the female reproductive tract and fetuses was assessed in immunodeficient (AG129) female mice that were exposed to ZIKV by subcutaneous (s.c.) inoculation, intravaginal (ivag.) inoculation, or sexual transmission from infected male AG129 mice. Pregnant females had significantly increased ZIKV dissemination to the female reproductive tract compared to non-pregnant females when exposed by s.c. or ivag. inoculation. Sexual transmission resulted in significantly greater morbidity and mortality in females and higher ZIKV titers in the female reproductive tract than s.c. or ivag. inoculation. Ovaries from females infected sexually contained ZIKV RNA within the ovarian follicles. Furthermore, ZIKV titers were significantly higher in fetuses from dams exposed sexually compared to fetuses from dams exposed by s.c. or ivag. inoculation. These results demonstrate that sexual transmission enhances dissemination of ZIKV to the female reproductive tract and developing fetuses in a mouse model.

## Introduction

Zika virus (ZIKV; *Flaviviridae*) is a mosquito-borne virus that is also transmitted sexually and *in utero*. ZIKV congenital syndrome occurs in fetuses or infants from at least 5% of infected pregnant women^1^, and disease outcomes vary in severity but can include microcephaly and other brain abnormalities, as well as eye, muscle and joint defects^2^. The route of infection (mosquito bite vs. sexual transmission) of pregnant women with fetuses or infants with ZIKV congenital infection is typically unknown. Some epidemiological studies have shown an increased incidence of ZIKV in women compared to men during outbreaks^3,4^, which may be due to male-to-female sexual transmission. ZIKV has been cultured from the semen of infected men^5,6^, and ZIKV RNA can be detected in semen from 30–50% of infected men in the first month after disease onset^7,8^. These findings indicate that ZIKV sexual transmission has the potential to occur frequently early after infection.

Vertical transmission of ZIKV has been shown by detection of the virus in fetal tissue. ZIKV RNA was detected in the placental or fetal tissue of 12% of pregnancies with possible maternal ZIKV exposure^9^. There have been several reports of prolonged ZIKV RNA in the serum of infected pregnant women for more than 2 weeks post-onset of disease^10–12^ or in urine for more than 4 weeks^13^, suggesting that immunosuppression from pregnancy or fetal infection may prolong maternal viremia; however, viremia in pregnant and non-pregnant women have not been directly compared. In rhesus macaque studies, ZIKV RNA was detected in serum for a longer duration in pregnant females compared to non-pregnant females^14,15^; however, another study showed persistent ZIKV RNA in the serum of non-pregnant rhesus macaques^16^.

Immunodeficient mice lacking all or part of the interferon response are more susceptible to ZIKV infection than wild-type mice due to the resistance of murine STAT-2 to ZIKV antagonism^17,18^ and are thus used for studies of ZIKV pathogenesis. Pregnant immunodeficient mouse models of ZIKV have shown *in utero* transmission following intravaginal inoculation^19^, subcutaneous inoculation^20^, and sexual transmission^21–23^. These and other studies of ZIKV infection in pregnant, immunodeficient female mice have demonstrated intrauterine growth restriction, fetal demise, fetal brain infection, and decreased cranial size of infected fetuses^24^. Transplacental infection of the fetus has been implicated by infection of placental trophoblasts in human placentas^25–28^ and pregnant mice^20^ and monocytes from human placentas^29^, though other studies have shown trophoblasts from human placentas to be resistant to ZIKV infection^30^. Infection of the maternal decidua has also been shown in pregnant rhesus macaques and in human placental explants^15,28^. Thus, the route of *in utero* transmission is still being investigated.

In order to assess the effect of the transmission route on the dissemination of ZIKV to the female reproductive tract and developing fetuses, female AG129 mice were inoculated by three different routes: subcutaneous inoculation (s.c.), intravaginal inoculation (ivag.), and sexual exposure. Pregnancy was found to increase ZIKV dissemination specifically to the female reproductive tract when females were inoculated s.c. or ivag. Infection by sexual exposure also increased dissemination to the reproductive tract in both non-pregnant and pregnant females, and to developing fetuses in pregnant females.

## Results

### Sexual transmission of ZIKV leads to more rapid disease in female AG129 mice

**Figure 1:**
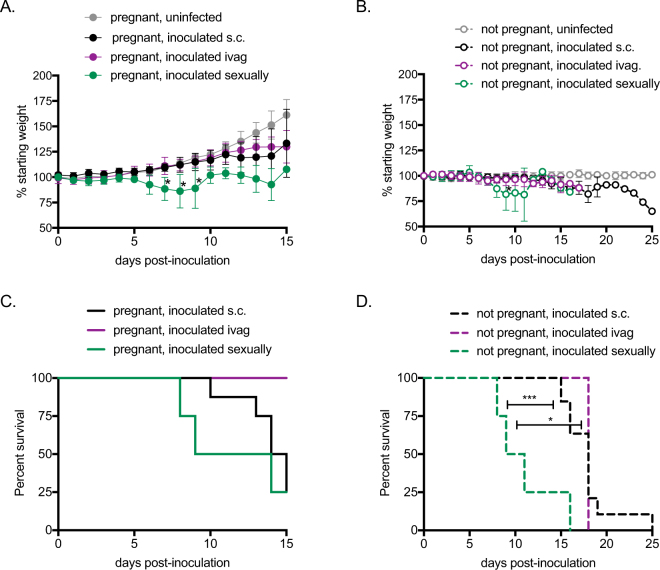
ZIKV disease by route of inoculation and pregnancy status. Gray symbols represent uninfected mice (n = 6 pregnant, n = 10 non-pregnant), black symbols represent mice inoculated s.c. (n = 8 pregnant, n = 13 non-pregnant), maroon symbols represent mice inoculated ivag. (n = 5 pregnant, n = 3 non-pregnant), and green symbols represent mice infected sexually (n = 4 pregnant, n = 4 non-pregnant). (**A**) Average weight of pregnant mice post-inoculation, represented as a percentage of initial weight. Error bars represent standard deviations. (**B**) Average weight of non-pregnant mice post-inoculation, represented as a percentage of initial weight. Error bars represent standard deviations. (**C**) Percent survival of pregnant mice post-inoculation. (**C**) Percent survival of non-pregnant mice post-inoculation. *p < 0.05, ***p < 0.001.

To assess the effect of pregnancy and route of inoculation on ZIKV pathogenesis, female AG129 mice were exposed to ZIKV strain PRVABC59 by three different routes. Pregnant and non-pregnant females were inoculated by subcutaneous (s.c.) or intravaginal (ivag.) routes 3 days after mating. Additional females were inoculated by mating to an infected male (sexually), which led to pregnancy in 40% of matings. For both the pregnant and non-pregnant groups, females that were infected with ZIKV sexually experienced more rapid and more severe weight loss than females that were infected via s.c. or ivag. routes (Fig. 1A, p < 0.05 dpi 6–8, and Fig. 1B, p < 0.05 dpi 9). Additionally, the median survival time was significantly shorter for non-pregnant females infected with ZIKV sexually compared to non-pregnant females infected via s.c. or ivag. routes (Fig. 1D, p < 0.001 and p < 0.05, respectively). Although not significant, the trend was similar for pregnant females, in which females infected with ZIKV sexually had a shorter mean survival time than females infected via s.c. or ivag. routes (Fig. 1C; p = 0.49).

### Inoculation route alters ZIKV kinetics in female AG129 mice

**Figure 2:**
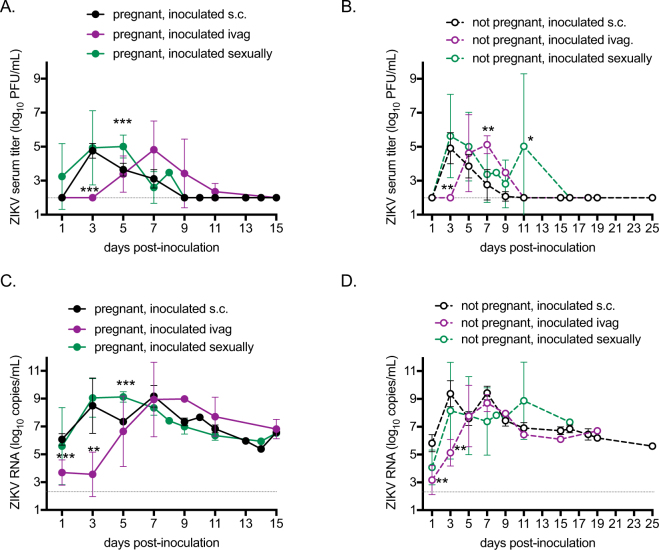
Viremia of ZIKV by route of inoculation and pregnancy status. Average viral titer and RNA copy number in serum. Black symbols represent mice inoculated s.c. (n = 8 pregnant, n = 13 non-pregnant), maroon symbols represent mice inoculated ivag. (n = 5 pregnant, n = 3 non-pregnant), and green symbols represent mice infected sexually (n = 4 pregnant, n = 4 non-pregnant). Error bars represent standard deviations. The limit of detection is represented by a dashed gray line. (**A**) ZIKV viremia in pregnant mice. (**B**) ZIKV viremia in non-pregnant mice (**C**) ZIKV RNA copy numbers in pregnant mice. (**D**) ZIKV RNA copy numbers in non-pregnant mice. *p < 0.05, **p < 0.01, ***p < 0.001.

Viremia profiles of females infected with ZIKV were assessed by plaque assay, and viral RNA was detected by qRT-PCR. Pregnancy status did not modulate viremia profiles of ZIKV for any inoculation route (Fig. 2A,B). However, viremia was delayed for females inoculated ivag., with mean peak titers occurring 4 days later than females inoculated sexually or by the s.c. route (Fig. 2A p < 0.001 dpi 3 and Fig. 2B, p < 0.01 dpi 3). In addition, viremias were prolonged for females inoculated sexually compared to females inoculated via s.c. or ivag. routes (Fig. 2A p < 0.001 dpi 5 and Fig. 2B, p < 0.05 dpi 11).

ZIKV RNA levels in serum reflected the patterns described above for viremia, in which the peak ZIKV RNA levels were delayed by 4 days in females inoculated by ivag. groups compared to females inoculated sexually or by the s.c. route (Fig. 2C). In addition, after infectious virus was cleared from the sera, ZIKV RNA was still detected at over 5 log_10_ ZIKV RNA copies/mL through the latest time points assessed for all groups. The persistent RNA in serum was found at indistinguishable levels in pregnant and non-pregnant females, indicating that prolonged ZIKV RNA in the serum was not pregnancy-dependent.

### Sexual transmission of ZIKV enhances dissemination to the female reproductive tract and fetuses

**Figure 3:**
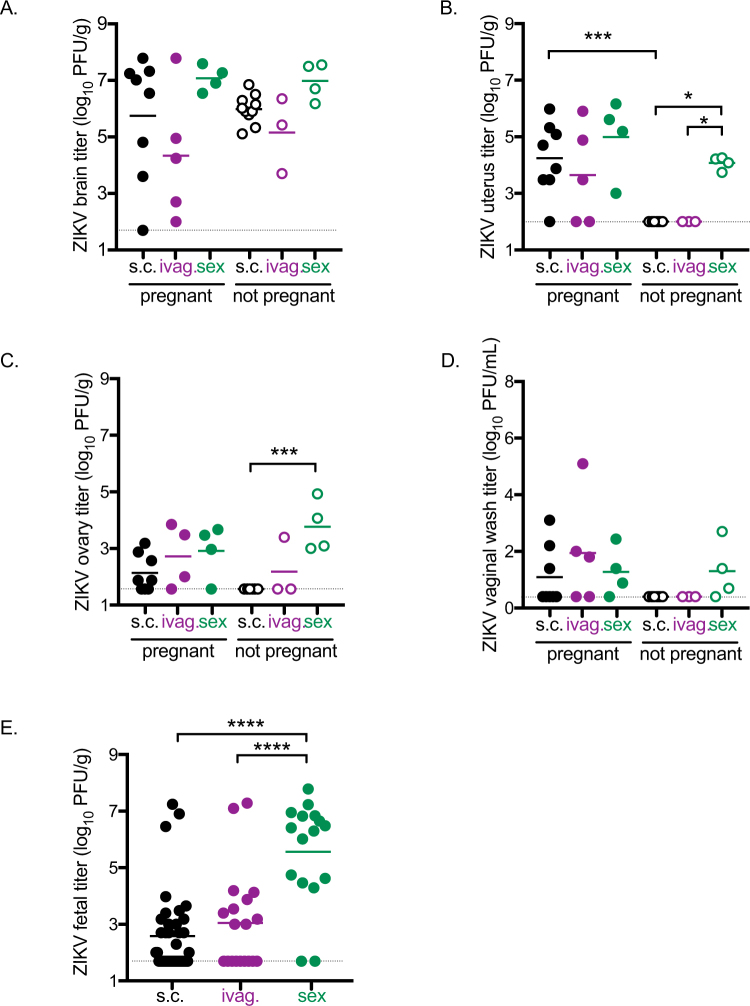
Tissue distribution of ZIKV by route of inoculation and pregnancy status. Black symbols represent mice infected s.c. (n = 8 pregnant, n = 13 non-pregnant), maroon symbols represent mice infected ivag. (n = 5 pregnant, n = 3 non-pregnant), and green symbols represent mice infected sexually (n = 4 pregnant, n = 4 non-pregnant).Titers from individual mice are represented by circles, with means represented by solid lines. The limit of detection is represented by a dashed gray line. (**A**) ZIKV titer in brain tissue. (**B**) ZIKV titer in uterine tissue. (**C**) ZIKV titer in ovaries. (**D**) ZIKV titer in vaginal washes. (**E**) ZIKV titer in fetal tissue. *p < 0.05, ***p < 0.001, ****p < 0.0001.

ZIKV titers were measured in maternal brain tissue, the female reproductive tract, and fetal tissues at the time of euthanasia. ZIKV titers in the brain were not significantly different between inoculation groups or by pregnancy status (Fig. 3A). For pregnant females, there were no significant differences in maternal tissue titers between any inoculation route. However, ZIKV titers in the uteri were significantly higher in non-pregnant females inoculated sexually compared to non-pregnant females inoculated s.c. or ivag. (Fig. 3B, p < 0.05) and in pregnant females compared to non-pregnant females for those inoculated s.c. (Fig. 3B, p < 0.001). Additionally, ZIKV titers in the ovaries were higher in non-pregnant females infected sexually compared to non-pregnant females infected via the s.c. route (Fig. 3C, p < 0.001) or the ivag. route (Fig. 3C, p = 0.74). The vaginal washes collected at the time of euthanasia also followed the same pattern of higher titers in non-pregnant females infected sexually compared to non-pregnant females infected via s.c. or ivag. inoculation, but titers were too low to establish significance (Fig. 3D). Thus, ZIKV disseminated to the upper and lower female reproductive tract in pregnant females irrespective of route of inoculation and in non-pregnant females only when exposed sexually.

Fetuses were collected from pregnant females at the time of euthanasia, and ZIKV titers were assessed. On average, 4.7 fetuses were collected per dam from 16 different dams. The percentage of fetuses that became infected was higher when the female was infected sexually (88%) compared to the percentage of fetuses from females infected via s.c. (50%) or ivag (53%) routes (p < 0.05). Titers were significantly higher in fetuses from females infected sexually compared to fetuses from females infected via s.c. or ivag routes. (Fig. 3E, p < 0.0001). The proportion of female mice with ZIKV-infected fetuses followed the same trend but was not significantly different between inoculation groups (100%, 63%, and 50% for sexual, s.c., and ivag. exposure routes, respectively). These data suggest ZIKV infection of fetuses occurs via both ascending and hematogenous routes.

### ZIKV RNA localizes to ovarian follicles and vasculature in females infected sexually

**Figure 4:**
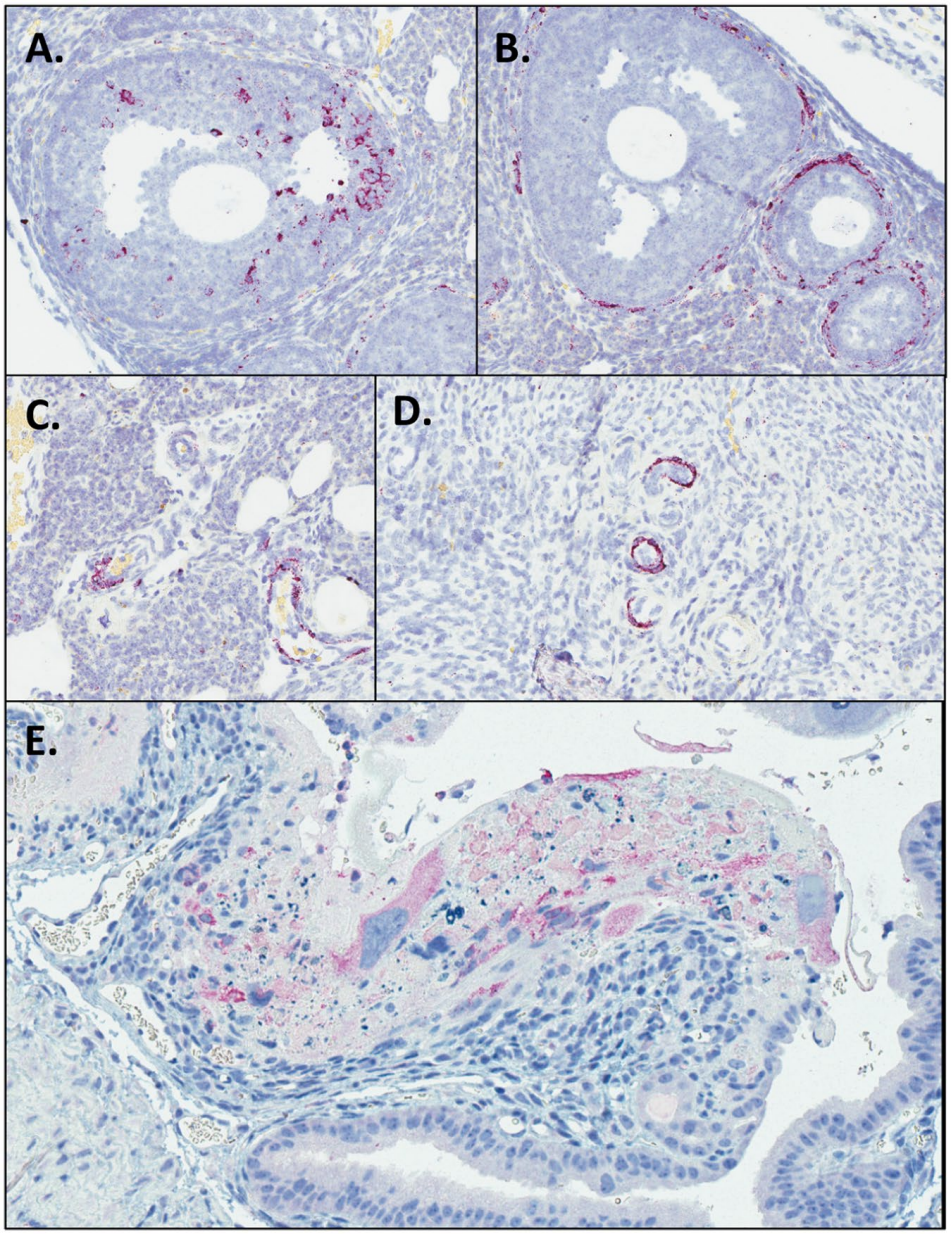
Localization of ZIKV in ovary and uterus of AG129 mice infected by sexual transmission. ZIKV RNA localization in ovarian granulosa cells (**A**), thecal cells (**B**), and ovarian vessels (**C**) by ISH. ZIKV RNA localization in uterine vessels by ISH (**D**). ZIKV antigen localization in trophoblasts of placental sites in uterus by IHC (**E**). Original magnifications: 200X (A–E).

In order to further assess the route of *in utero* transmission and whether infection of the ovary may contribute to decreased fertility of infected females, ZIKV RNA and antigen were localized within tissues of females infected sexually using *in situ* hybridization (ISH) and immunohistochemistry (IHC). Tissues from females infected by the s.c. or ivag. route were not assessed. By ISH, ZIKV nucleic acid was detected within the theca interna of the perifollicular stroma and within vascular endothelium in the ovaries from 6 out of 7 infected females, and within scattered granulosa cells of the ovarian follicles from 2 out of 7 females (Fig. 4A–C). One of the two also showed ZIKV antigen in scattered granulosa cells by IHC. The two females with follicular granulosa cell staining were euthanized earliest on dpi 8, and one of the two females was pregnant. These results suggest that ZIKV infection of the ovarian follicles occurs early. Furthermore, ZIKV was detected by ISH within the uterine vasculature of both non-pregnant and pregnant females and in association with neutrophilic inflammation in the myometrium of one non-pregnant animal, and within placental sites, including trophoblasts, of pregnant females by ISH and IHC (Fig. 4D,E).These findings suggest the potential for transplacental ZIKV transmission to the fetus, as well infection from the maternal decidua.

## Discussion

Sexual transmission of ZIKV to female AG129 mice increased the infection rate and viral titer in developing fetuses compared to s.c. or ivag. inoculation routes (Fig. 3). In addition, dissemination of ZIKV to the female reproductive tract was enhanced for non-pregnant females following sexual transmission. While pregnancy increased ZIKV titers in the female reproductive tract following inoculation by s.c. or ivag. routes, the effect of pregnancy on viral dissemination was not evident when females were infected sexually, suggesting that an ascending infection is a more efficient route of infection in the absence of pregnancy. In this mouse model, these results indicate that sexual transmission of ZIKV alters the dissemination of the virus, leading to more frequent *in utero* transmission.

Intravaginal inoculation of pregnant mice and sexual transmission have previously been shown to lead to fetal infection^19,21^. Female macaques are also susceptible to intravaginal ZIKV inoculation^31^, and intravaginal ZIKV inoculation of rhesus macaques leads to altered kinetics compared to subcutaneous inoculation^32^, similar to the results presented here. Previous studies have shown that hormone treatment alters susceptibility to intravaginal ZIKV inoculation in macaques and mice^32,33^, and may thus alter dissemination of the virus in the female reproductive tract.

Prolonged ZIKV RNA in blood has been reported in a few cases of infected pregnant women^10–12^; however, the results presented here show that ZIKV RNA clearance from the blood is similar in pregnant and non-pregnant female mice and was therefore not dependent on pregnancy status in this model. The results here do show that pregnancy may increase the dissemination of ZIKV to the female reproductive tract after s.c. or ivag. inoculation, perhaps because pregnancy decreases the cellular adaptive immune response in mice infected with ZIKV^34^. However, we have shown here that ivag. inoculation does not accurately mimic sexual transmission, perhaps because in mice sex results in deposition of ejaculates directly or nearly directly into the uterus^35,36^. Components of seminal plasma or cell-associated virus, which are absent in ivag. inoculation, may also be responsible for the enhanced distribution of ZIKV after sexual transmission. The inflammation induced in the female reproductive tract by semen^37,38^ may impact susceptibility to viral infection, or seminal amyloids such as semen-derived enhancer of viral infection (SEVI), which have been shown to enhance HIV infection^39^, may play a role with ZIKV pathogenesis in the female reproductive tract as well. Some caveats to these results are that viral dose cannot be controlled during sexual transmission and that infection through the sexual route occurred simultaneously with pregnancy. However, previous studies demonstrated that titers of 10^3^ PFU per ejaculate were common during the infectious period of seminal shedding in inoculated male AG129 mice^21,40^. Simultaneous pregnancy and infection through the sexual route could explain the failure to identify a significant enhancing effect of pregnancy on titers in the female reproductive tract after sexual transmission.

ZIKV RNA was detected within ovarian follicles of mice exposed to ZIKV sexually. Whether ZIKV infection can alter female fertility is unknown, though in male mice ZIKV testicular infection causes testicular atrophy^41,42^, and a study on male fertility using small numbers of human samples showed a modest, transient decrease in total sperm after ZIKV infection^43^. Further studies of the effect of ZIKV infection on ovulation and female fertility are warranted.

The results presented here suggest that sexual transmission may play an important role in ZIKV infection during pregnancy and the development of fetal ZIKV congenital syndrome. Sexual transmission of ZIKV may alter the tropism and/or dissemination of the virus throughout the female reproductive tract compared to mosquito-borne transmission in both pregnant and non-pregnant women. Understanding how sexual exposure could alter dissemination of ZIKV may help to explain the mechanisms of *in utero* ZIKV transmission to a developing fetus and provide a scientific foundation for assessing risk factors for sexual transmission to reduce congenital ZIKV infections.

## Materials and Methods

### Inoculation of AG129 mice

Interferon α/β and –γ receptor knockout AG129 mice were originally obtained from B&K Universal (Hull, United Kingdom) and bred in-house. Thirty-nine female AG129 mice were mated to uninfected male AG129 mice. Three days after a copulatory plug was identified, females were inoculated with 10^3^ PFU of ZIKV isolate PRVABC59 either through the s.c. route in the footpad (n = 11) or through the ivag. route (n = 28), as described previously^21^. Twenty-six mice were not mated and were inoculated on a random day of their estrous cycle by the s.c. route (n = 10) or through the ivag. route (n = 16). Ten additional female AG129 mice were mated to male AG129 mice beginning 7 days after the males were inoculated s.c. with 10^3^ PFU to maximize exposure^40^, until observation of a copulatory plug as evidence of mating, which took a maximum of 6 days. Mice were euthanized by isoflurane-induced deep anesthesia followed by cervical dislocation at gestational day 18 or when clinical evidence of disease was observed. Tissues, serum, and vaginal washes were collected at the time of euthanasia. Tissue samples were weighed, and an equal volume of BA-1 medium was added before homogenization using a pestle, followed by centrifugation to clarify samples. Viral titers were measured by Vero cell plaque assay^21^. All animal studies were conducted under approved IACUC protocols at the Centers for Disease Control and Prevention. All protocols and practices for the handling and manipulation of mice were in accordance with the guidelines of the American Veterinary Medical Association (AVMA) for humane treatment of laboratory animals.

### ZIKV RNA quantification

RNA was extracted from serum samples using the MagMAX Viral RNA isolation kit (Ambion). ZIKV RNA was quantified using real-time RT-PCR primers and probe as described previously^44^. A standard curve was generated by *in vitro* transcription of a plasmid containing a fragment of ZIKV spanning nucleotides 859–1278 as described previously^21^. The detection limit for this assay was 2.3 log_10_ RNA copies/mL.

### Histology, IHC, and ISH

Tissues were placed into 10% neutral buffered formalin for 3 days and then stored in 70% ethanol prior to processing. Sections were cut at 4 microns and stained with hematoxylin and eosin or by *in situ* hybridization (ISH) for ZIKV RNA. The ISH assay was performed using the ViewRNA ISH tissue assay (ThermoFisher) with a probe set targeting the ZIKV (+) strand RNA genome. As a negative control, tissue sections from an uninfected female mouse were stained using the ZIKV probe set. As a positive control, tissues were stained using probes against the housekeeping murine mRNAs GAPDH, PIPB, and β-actin. Tissue sections were de-paraffinized and underwent pre-treatment and protease treatment for ten minutes each. Positive staining (red punctate staining) was detected using an alkaline-phosphatase label probe. The slides were counterstained with Gill’s Hematoxylin I. The IHC assay was performed using a polymer-based indirect immunoalkaline phosphatase detection system with colorimetric detection of antibody/polymer complex with Fast Red chromogen. The primary antibody was a rabbit polyclonal antibody generated against ZIKV VLPs^21^. Negative controls for IHC comprised both uninfected tissues incubated with the primary antibody, and infected tissues incubated with normal rabbit serum in place of the primary antibody.

### Statistical analyses

ZIKV RNA, viremia, and weights were compared using t-tests with a correction for multiple comparisons. ZIKV titers in tissues were compared using ANOVA with a correction for multiple comparisons. Proportions were compared using Fisher’s exact test. Survival curves were compared using a logrank test. All tests were performed in Prism7.
